# Single-molecule measurements reveal that PARP1 condenses DNA by loop stabilization

**DOI:** 10.1126/sciadv.abf3641

**Published:** 2021-08-11

**Authors:** Nicholas A. W. Bell, Philip J. Haynes, Katharina Brunner, Taiana Maia de Oliveira, Maria M. Flocco, Bart W. Hoogenboom, Justin E. Molloy

**Affiliations:** 1 The Francis Crick Institute, London NW1 1AT, UK.; 2 London Centre for Nanotechnology, University College London, London WC1H 0AH, UK.; 3 Molecular Sciences Research Hub, Department of Chemistry, Imperial College London, London W12 0BZ, UK.; 4 Department of Physics and Astronomy, University College London, London WC1E 6BT, UK.; 5 Discovery Biology, Discovery Sciences, R&D, AstraZeneca, Cambridge, UK.; 6 Mechanistic and Structural Biology, Discovery Sciences, BioPharmaceuticals R&D, AstraZeneca, Cambridge, UK.

## Abstract

Poly(ADP-ribose) polymerase 1 (PARP1) is an abundant nuclear enzyme that plays important roles in DNA repair, chromatin organization and transcription regulation. Although binding and activation of PARP1 by DNA damage sites has been extensively studied, little is known about how PARP1 binds to long stretches of undamaged DNA and how it could shape chromatin architecture. Here, using single-molecule techniques, we show that PARP1 binds and condenses undamaged, kilobase-length DNA subject to sub-piconewton mechanical forces. Stepwise decondensation at high force and DNA braiding experiments show that the condensation activity is due to the stabilization of DNA loops by PARP1. PARP inhibitors do not affect the level of condensation of undamaged DNA but act to block condensation reversal for damaged DNA in the presence of NAD^+^. Our findings suggest a mechanism for PARP1 in the organization of chromatin structure.

## Teaser

A combination of single-molecule methods shows that PARP1 condenses double-stranded DNA by a loop stabilization mechanism.

## INTRODUCTION

The poly(ADP-ribose) polymerase (PARP) family of enzymes catalyze the transfer of ADP-ribose units to target proteins. The human genome encodes 17 PARP family genes, which play a wide variety of roles in cellular processes (*1*, *2*), with PARP1 accounting for 90% of ADP-ribose synthesis (*3*). Since the 1980s, it has been known that the catalytic activity of PARP1 is stimulated by binding at single-strand breaks (SSBs) and double-strand breaks in DNA (*4*). This activity is a key stage in single-strand DNA break repair, with the formation of poly(ADP-ribose) (PAR) acting to loosen chromatin (*5*) and to recruit additional DNA repair factors (*6*, *7*). Much recent work on analyzing PARP1 has been stimulated by the discovery that inhibition of PARP activity results in synthetic lethality toward breast cancer gene (BRCA)–mutated cancer cells (*8*, *9*). PARP inhibitors trap the protein at sites of DNA damage by inhibiting enzymatic activity and thereby block PAR-induced unbinding (*10*–*13*). This leads to increased double-strand breaks via collapse of replication forks (*14*, *15*) and confers sensitivity of BRCA-mutated cells to PARP inhibitor drugs.

PARP1 is a multidomain protein with three zinc fingers at its N-terminus followed by an automodification domain, a DNA binding WGR-motif domain and a catalytic domain at the C-terminus (*16*). The first two zinc fingers coordinate binding at a single-strand DNA break by forming contacts between the exposed bases and hydrophobic residues of the protein (*17*, *18*). By binding at a site of DNA damage, PARP1 initiates the assembly of its multiple domains into a compact structure (*19*). This results in the unfolding of specific autoinhibitory structures in the catalytic domain, thereby enabling access of nicotinamide adenine dinucleotide (NAD^+^) to the active site and subsequent PAR synthesis (*20*, *21*). Biochemical studies have shown that PARP1 has a high affinity for DNA lesions and a moderate affinity for undamaged DNA. For PARP1 binding to double-strand breaks or SSBs at physiological salt concentrations, typical values for *K*_d_ (dissociation constant) are in the range of 10 to 100 nM (*21*–*23*). The affinity of PARP1 for an 18–base pair (bp), ligated, symmetric dumbbell, which mimics undamaged DNA, was measured as *K*_d_ = 1 μM at 100 mM NaCl concentration (*17*). Studies on the dynamics of PARP1 show that it rapidly accumulates at sites of DNA damage in the nucleus (*24*) using intersegmental transfer along DNA to increase the speed of damage localization (*25*).

Together with its role in DNA repair, PARP1 acts as a chromatin architectural protein and transcriptional regulator (*26*–*28*). PARP1 binds tightly to nucleosome-decorated DNA and represses transcription (*29*, *30*). Depletion of PARP1 by inducible antisense RNA substantially increases the susceptibility of HeLa cell chromatin to nuclease degradation (*31*, *32*). There are on the order of a million PARP1 molecules per cell (*33*), which is equivalent to approximately 1 PARP1 molecule per 20 nucleosomes (*22*), making it one of the most abundant nuclear proteins, with a nuclear concentration of ~20 μM. This statistic underlines the potential of PARP1 to shape chromatin architecture. In addition to its enzymatic activation by DNA damage, PARP1 can also be activated by various environmental cues such as heat shock, which causes increased gene expression (*34*). Histone modifications are likely activators in this context (*35*–*37*). Overall, the multifunctional roles of PARP1 motivate a need to increase our understanding of its DNA binding modes and the effects of therapeutic drugs on PARP1-DNA interactions.

Here, we have used a combination of single-molecule approaches to measure PARP1-DNA structural dynamics in real time. We find that PARP1 causes compaction of both damaged and undamaged DNA at sub-piconewton tension and that high forces result in stepwise unbinding of PARP1. We also find that PARP1 can bridge two separate DNA molecules that are brought in close proximity by DNA braiding. These observations lead us to propose a loop stabilization model where PARP1 can bridge the intersection of two DNA double strands. The presence of DNA damage and NAD^+^ causes rapid destabilization of DNA loops—an effect which is blocked by catalytic site inhibitors.

## RESULTS

### Fluorescence microscopy demonstrates condensation of DNA by PARP1

**Fig. 1:**
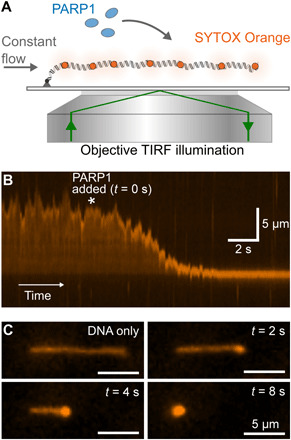
TIRF imaging shows condensation of DNA by PARP1. (**A**) Schematic of TIRF microscopy imaging of a single λ-DNA molecule stained with SYTOX Orange. A constant flow was maintained, which stretches out the DNA over a distance close to its contour length. (**B**) Kymograph showing DNA extension over time. 400 nM PARP1 is added at the time point indicated by the asterisk. (**C**) Snapshots showing individual image frames at the indicated time points.

PARP1 binding to long stretches of undamaged DNA was visualized via total internal reflection fluorescence (TIRF) video microscopy using SYTOX Orange–labeled λ-DNA (48.5 kbp long). The DNA was bound at one end to the microscope coverslip via a biotin-streptavidin linkage; continuous solution flow, applied with a syringe pump (Fig. 1A), stretched out the molecule to approximately 70% of its ~16-μm contour length. Before PARP1 addition, the DNA exhibited length fluctuations due to thermal forces; after addition of 400 nM PARP1, the DNA condensed toward its attachment point on the surface (Fig. 1B and fig. S1 for full field of view). Condensation commenced at the free end of the molecule, where a compact structure initially formed and then gradually increased in intensity as it moved toward the anchor point (Fig. 1C). Note that the hydrodynamic flow stretches DNA molecules with a line tension that decreases from a maximum value at the molecule’s attachment point to zero at the free end (*38*, *39*). Hence, while our TIRF results clearly demonstrate that DNA condensation by PARP1 is initiated at the free end, it is not possible to distinguish whether this is caused by the presence of a double-strand break or the lower tension toward the free end.

### AFM shows decoration of undamaged DNA by single PARP1 molecules

**Fig. 2:**
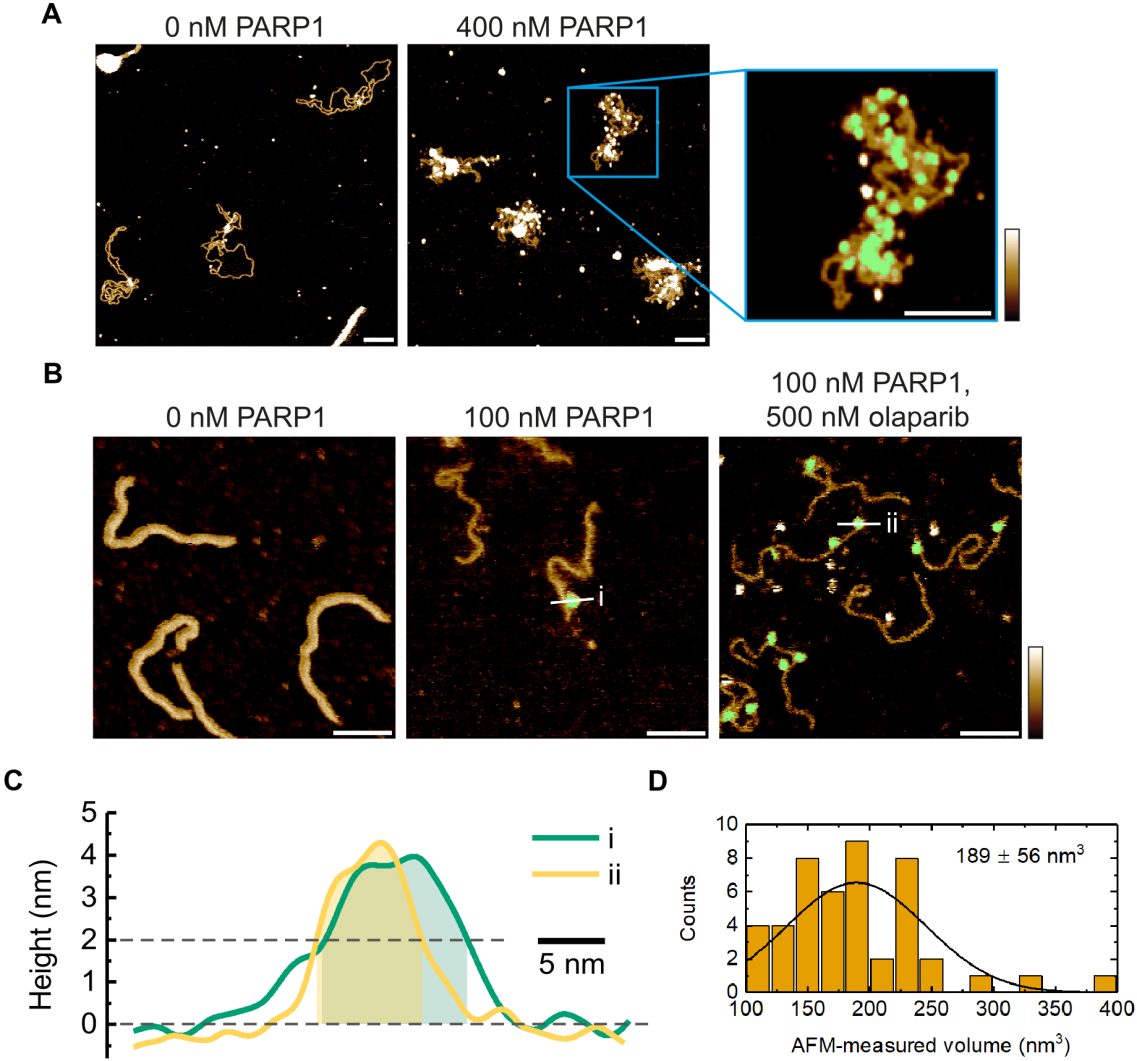
AFM characterization of PARP1-DNA binding. (**A**) AFM images of PARP1 binding to a 4.4-kbp, covalently closed plasmid. Inset: Zoom-in image showing PARP1 bound to plasmid DNA. The presence of bound PARP1 is indicated in green, representing the pixels where the local height exceeded a threshold of ~2 nm above the background. Scale bars, 100 nm. Color scale, 2.5 nm. (**B**) Images showing PARP1 binding to a 496-bp polymerase chain reaction (PCR) fragment with a SSB approximately one-third of the way along the contour length. The green indicates bound PARP1 that was selected for volume estimation. Scale bars, 50 nm. Color scale, 2.5 nm. (**C**) Two selected height profiles with corresponding proteins shown in (B). We observe similar height profiles for PARP1 molecules bound to DNA in the presence and absence of 500 nM olaparib. The transparent areas indicate the masked portion of the profiles that contributed to the volume estimate. (**D**) Histogram of observed PARP1 volumes when bound to the 496-bp PCR fragment.

To understand the nature of PARP1 binding to stretches of undamaged DNA, we visualized PARP1-DNA complexes by atomic force microscopy (AFM) in liquid. First, we incubated PARP1 with a 4.4-kbp relaxed, covalently closed circular plasmid, i.e., an undamaged DNA molecule. The sample was next adsorbed at a mica surface that was functionalized with poly-l-lysine–poly(ethylene) glycol (PLL-PEG) to adhere the DNA while also minimizing nonspecific surface adsorption of the PARP1 (*40*). This resulted in extensive decoration of the plasmids with PARP1 molecules, appearing as bright dots on top of the DNA (Fig. 2A), with no apparent preference for any specific location along the DNA. By comparison of multiple images, we found that this decoration did not result in significant compaction of the plasmid, probably due to the fact that the extension of DNA in these experiments may be largely determined by its binding to the AFM substrate, trapping it in nonequilibrated, compacted conformations independent of protein binding (*41*).

For comparison, we also imaged PARP1 binding to a short, 496-bp polymerase chain reaction (PCR) fragment that contained a SSB site one-third of the way along its contour length in the absence and presence of a PARP1 inhibitor (olaparib). As shown in Fig. 2B, we observed bright dots, attributed to PARP1, at locations that were consistent with its binding not only to the SSB but also to undamaged sections of the DNA in agreement with previous AFM results on dried samples (*42*,*43*). By considering the local height of dots above the substrate (Fig. 2C), we calculated the observed volume distribution (Fig. 2D), which had a median of 182 nm^3^. From the crystal structure of PARP1, we estimated an equivalent spherical volume of 150 nm^3^ (fig. S2). Therefore, the images of isolated spots are consistent with a monomer of PARP1. This agrees with previous AFM measurements of PARP1 (*43*), also when taking into account the possible effects of the AFM tip on the appearance of the molecule (fig. S2).

This volume measurement also facilitated the quantification of the number of bound PARP1 molecules on the plasmid DNA. To this end, we created a mask that defined areas of PARP1 binding (Fig. 2A, magnified section) and then calculated the total volume under the mask before dividing by the volume of a single PARP1 molecule. Analysis of multiple images yielded estimates of the number of bound PARP1 molecules of 16 ± 3 (means ± SE, *n* = 18) at 200 nM PARP1 and of 59 ± 15 (means ± SE, *n* = 13) at 400 nM PARP1 (fig. S3). Last, to verify the robustness of our observations against variations in sample preparation, we also analyzed DNA that was exposed to PARP1 after DNA adsorption at the AFM substrate. While this resulted in lower amounts of bound PARP1, it yielded qualitatively similar results (figs. S4 and S5).

### Real-time measurement of DNA condensation by magnetic tweezers

**Fig. 3:**
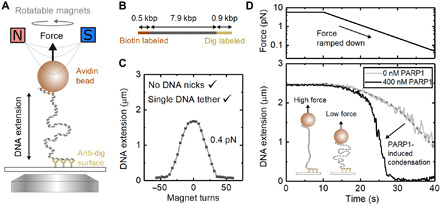
Magnetic tweezers showing PARP1-induced condensation on undamaged DNA. (**A**) Schematic of magnetic tweezers showing DNA stretched between the bead and coverslip. The height of the magnets controls the force acting on the bead, and the bead can be rotated by rotating the magnets. (**B**) DNA construct used for magnetic tweezers with two labeled handles acting as attachment points. (**C**) Single DNA tethers without nicks are selected by examining the characteristic response of DNA extension to magnet turns. (**D**) Effect of force ramp on DNA extension in the presence of 400 and 0 nM PARP1. At low forces (<1 pN), PARP1 induces significant compaction of the DNA. The dashed gray line shows a fit of the worm-like chain model to the DNA extension for 0 nM PARP1 with a fixed persistence length of 50 nm. The DNA contour length was a free parameter for the fit and yielded 2.7 μm in good agreement with the expected length of 2.6 μm for the 7.9-kbp DNA.

**Fig. 4:**
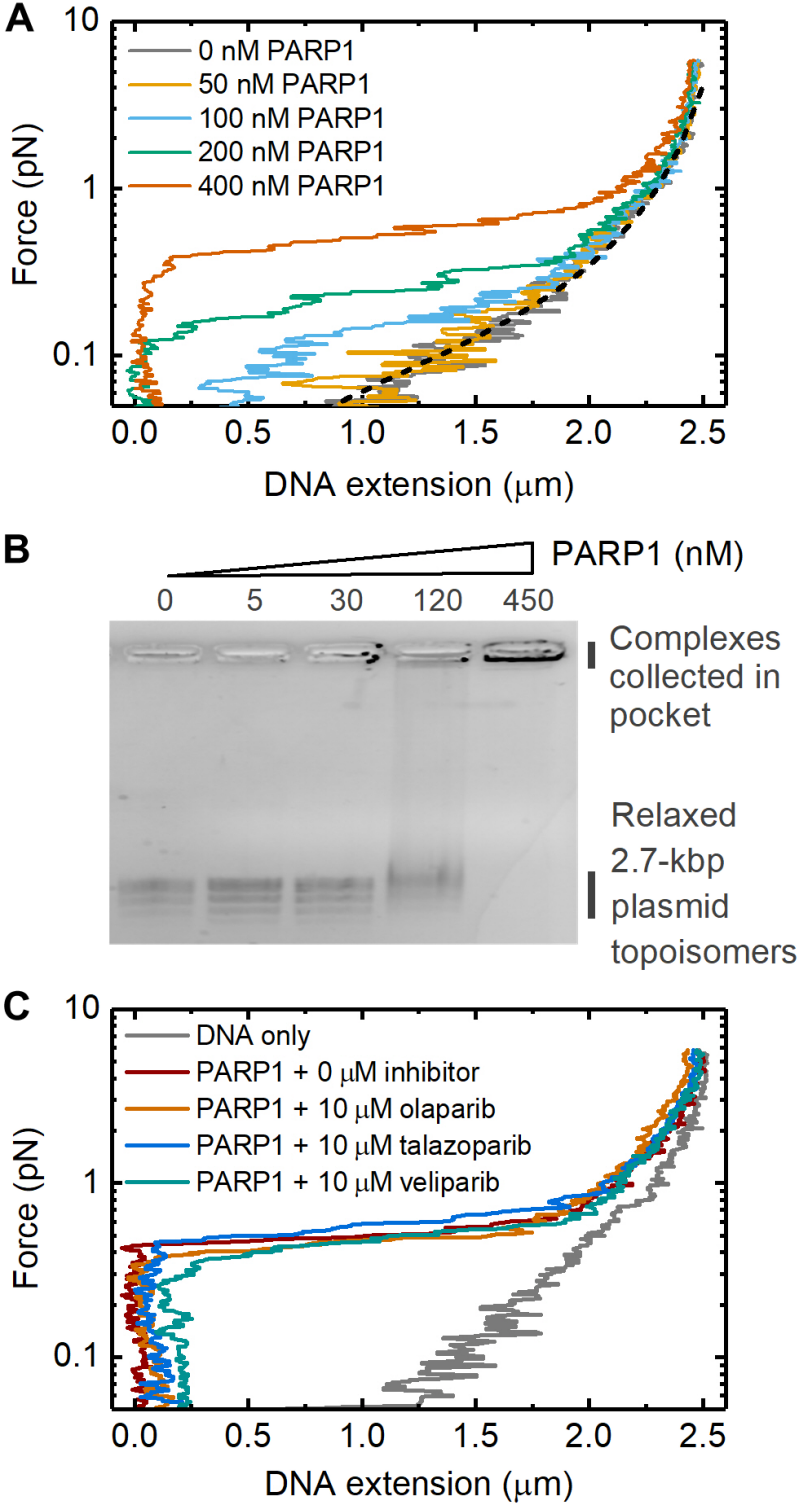
Concentration dependence of DNA condensation by PARP1. (**A**) Magnetic tweezers data showing the change in DNA extension at different applied forces. The dashed black line indicates the worm-like chain model prediction for naked DNA. (**B**) Agarose gel electrophoresis showing titration of PARP1 with 3 nM relaxed, covalently closed 2.7-kbp DNA plasmid. Several bands are visible for the plasmid sample, corresponding to different topoisomers. (**C**) Force-extension curves measured in the presence and absence of three PARP inhibitors. 400 nM PARP1 concentration was used in each case.

Noting the PARP1-induced condensation of DNA as observed by TIRF microscopy and the overall decoration of undamaged DNA by bound PARP1 molecules in the AFM images, we next sought to characterize the PARP1-induced condensation with precise control of tension and DNA damage using magnetic tweezers. We tethered individual DNA molecules (7.9 kbp in length) to a magnetic bead and applied controlled forces and rotations via a pair of permanent magnets (Fig. 3, A and B). The DNA extension was measured by real-time video tracking of the bead position, while the force was controlled by varying the separation between the bead and the magnets. The use of magnetic tweezers enabled us to verify that there was a single, undamaged DNA molecule tethered between the surface and bead based on the characteristic change in DNA extension upon bead rotation at low force (Fig. 3C) (*44*). In addition, as the applied tension (force) was reduced from 5.8 to 0.07 pN (Fig. 3D), DNA extension in the absence of PARP1 decreased in a manner consistent with the worm-like chain force-extension property that is typical for single DNA molecules (see Fig. 4A) (*45*).

While the addition of 400 nM PARP1 made little difference to DNA length measured at higher forces (Fig. 3D), it caused a marked shortening of the DNA as force was reduced below 1 pN, consistent with our observation by TIRF microscopy. To confirm that the observed compaction was specific to PARP1 and not due to any copurifying contaminants, we performed a control experiment that showed no DNA compaction when PARP1 was specifically depleted by immunoprecipitation (fig. S6). We also repeated the experiment using the same (48.5-kbp) λ-DNA template, as used in our previous TIRF experiments, and confirmed the same force-extension behavior with DNA compaction occurring at forces below 1 pN in the presence of 400 nM PARP1 (fig. S7). Taking these results together, we conclude that PARP1-induced condensation does not depend on the presence of a free end or double-strand break (as in the TIRF experiments in Fig. 1) but does strongly depend on the DNA tension.

The condensation of undamaged DNA also showed a strong dependence on PARP1 concentration, as measured via force-extension curves at different PARP1 concentrations (Fig. 4A). The force was ramped using the same profile as shown in Fig. 2D. DNA condensation was observed at concentrations of 100 nM PARP1 and above, and, for increasing PARP1 concentration, higher forces were required to extend the DNA to lengths observed in the absence of PARP1. To characterize the condensation at effectively zero force, we analyzed binding to relaxed, covalently closed plasmid DNA by agarose gel electrophoresis. This showed the formation of PARP1-DNA complexes at 120 nM PARP1 and above, as indicated by a smearing of the DNA plasmid bands (Fig. 4B). At 450 nM PARP1 concentration, the complexes did not run through the gel and collected in the gel pocket. We also measured the effect of several PARP inhibitors on the condensation of undamaged DNA (Fig. 4C). At 10 μM concentration of each of olaparib, talazoparib, and veliparib, we observed no effect on PARP1-induced DNA condensation. By contrast, experiments with PARP2 showed no condensation effect at concentrations up to 5 μM (fig. S8). Recent crystal structures show that PARP2 is able to bridge double-strand breaks between two nucleosomes (*46*). Our assay, however, shows that there is no associated bridging on intact DNA. We also conducted experiments with the first two zinc fingers of PARP1 (Zn1Zn2), which showed no condensation at concentrations up to 5 μM (fig. S9).

### Magnetic tweezers reveal stepwise unbinding and DNA bridging by PARP1

**Fig. 5:**
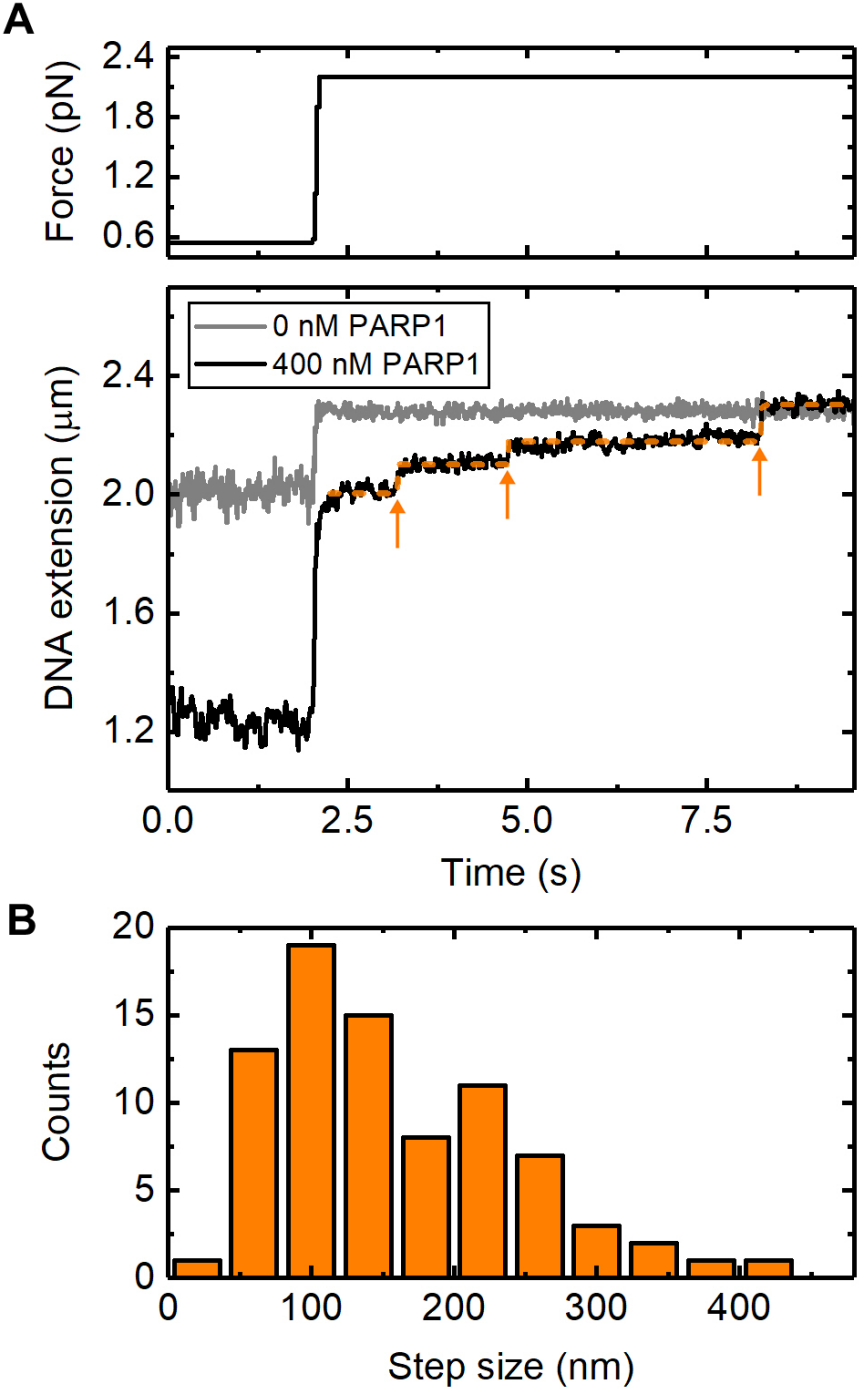
Stepwise reversal of PARP1-induced condensation, as observed upon application of high forces. (**A**) Traces for 0 and 400 nM PARP1, showing the changes in DNA extension after a step change in force from 0.6 to 2.2 pN. The orange line indicates the result of the step-fitting algorithm used with the arrows showing the positions of individual detected steps. A delay of 0.2 s between the force change and the beginning of step detection was imposed. (**B**) Histogram of the measured step sizes. Eighty-one steps were detected from a total of 44 traces where the force was stepped from 0.6 to 2.2 pN.

To gain further insight into the mechanism of PARP-induced DNA condensation, we examined the reversal of condensation by monitoring the time course of DNA extension changes following the application of a rapid increase in force applied to the magnetic bead. Figure 5A shows an example trace where we applied a step change in force from 0.6 to 2.2 pN and measured the resulting response in DNA extension over time. In the absence of PARP1, a step change in force produces a rapid change in DNA extension. In the presence of PARP1, however, we observed a different behavior. Specifically, DNA extension showed initially a rapid change in length followed by a series of discrete steps to reach its final extension (marked by arrows in the bottom panel of Fig. 5A). We collected 44 traces and used a chi-squared minimization method to detect the time and amplitude of the stepwise length changes following the force step (*47*, *48*). Figure 5B shows a histogram of the measured step amplitudes with an average step size of 158 ± 10 nm (mean ± SE).

The observed step sizes are two to five times larger than the approximate circumference of PARP1 (see fig. S2), arguing against a model where DNA is tightly wrapped around PARP1, such as in a nucleosome. Instead, the large step amplitude, together with the sub–1-pN threshold for condensation (Fig. 4A), suggests that PARP1 stabilizes loops of DNA that are formed under thermal fluctuations. The statistical mechanics of DNA under tension shows that DNA loops, formed by thermal fluctuations, are strongly suppressed when force is applied to DNA (*49*, *50*). This sub-piconewton force threshold is in agreement with other proteins which are known to stabilize DNA loops (*51*, *52*). Worm-like chain models of DNA force-extension behavior indicate that the expected distribution of loop sizes will be centered at several times the 50-nm persistence length of DNA with a range of several hundred nanometers (*50*). When the DNA is under zero tension, such as the conditions used for agarose gel electrophoresis analysis (Fig. 4B), the loop-size distribution is a competition between the enthalpic cost of forming tight DNA bends, disfavoring short loops and the entropic cost of bringing two distant regions of DNA close enough to intersect, which disfavors long loops. Applying tension to the DNA introduces an extra work term into the free energy such that the likelihood of loop formation is extremely low (above ~1 pN) (*49*).

**Fig. 6:**
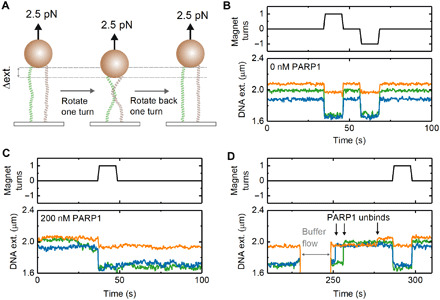
Bridging of two DNA molecules by PARP1. (**A**) Schematic of experiment—rotating a dual-tethered magnetic bead results in the formation of a DNA braid and a change in measured extension. (**B**) In the absence of PARP1, rotation of the magnetic bead clockwise (+1 turns) or counterclockwise (−1 turns) results in a reversible change in DNA extension. The three colors represent the data recorded simultaneously from three beads in the field of view. (**C**) In the presence of 200 nM PARP1, the effect of a one-turn rotation is found to be irreversible, until (**D**) the flow through of 0 nM PARP1 buffer (at *t* = 230 s) results in a rapid reversal to original DNA extension. Arrows indicate steps where DNA extension returns to original value.

A DNA looping mechanism requires that two sections of DNA are bridged where they intersect. To test whether such a mechanism might apply to PARP1-induced DNA condensation, we investigated the ability of PARP1 to bind at the intersection of two double-stranded DNA (dsDNA) strands. In these experiments, the surface density of DNA on the magnetic beads was increased by incubation with a higher concentration of DNA. This resulted in a significant number of beads that were connected to the microscope coverslip by two DNA molecules. A single rotation of such, doubly tethered, beads causes a braid to form between the two DNA molecules, which leads to a marked decrease in DNA extension, as observed via the decrease in bead height (Fig. 6A) (*53*, *54*). In a typical field of view, the majority of beads were still bound via a single DNA tether giving confidence that attachment via three or more tethers was rare. The size of the height decrease resulting from a single-bead rotation is dependent on the exact position of the anchoring points of the two tethers on the bead and surface. In the absence of protein, braid formation is reversible and can be unwound simply by rotating the bead back to its starting position. Figure 6B presents data traces acquired using three independent beads imaged in the same experiment. Each bead showed the characteristic change in extension upon rotation in the absence of PARP1. In the presence of 200 nM PARP1, we observed that the extension was stabilized at the braided level when the magnets are first rotated by one turn to form the braid and then rotated back to the start position (Fig. 6C). This shows that PARP1 bridges the two DNA molecules at their crossover point and prevents them from coming apart when the two strands are unwound. After flowing through buffer to remove free PARP1 from the flow cell (Fig. 6D), we observe rapid unbinding of the PARP1 as shown by the unwinding of the DNA braids in less than 30 s.

### Kinetics of condensation loss in the presence of DNA damage and NAD^+^

**Fig. 7:**
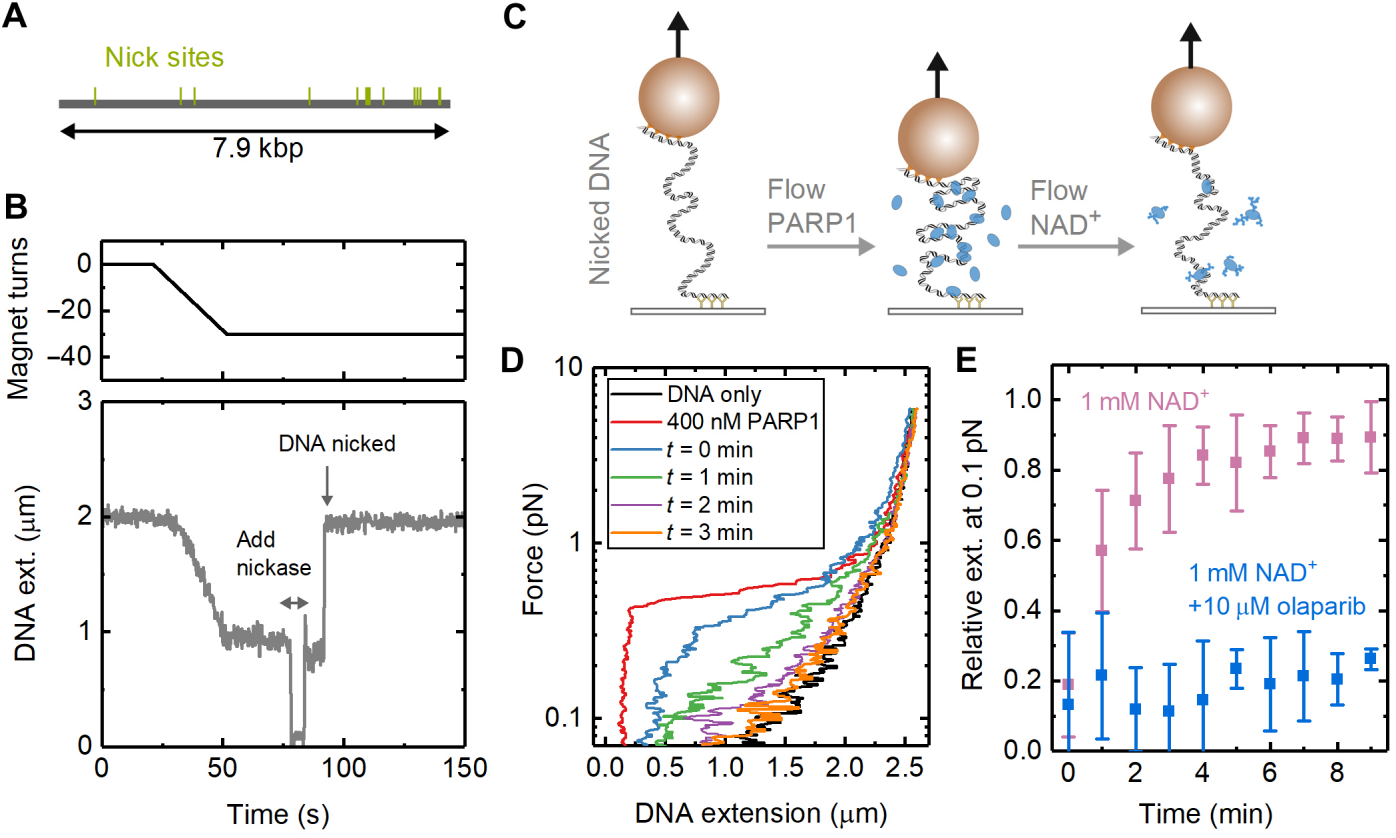
Reversal of PARP1-induced DNA condensation in the presence of NAD^+^ and DNA damage. (**A**) Positions of 14 nick sites on 7.9-kbp DNA section of magnetic tweezers for the nicking endonucleases Nt.BsmAI and Nb.BsmI. (**B**) Example trace showing the formation of nicks visualized in real time with magnetic tweezers. The DNA is coiled by magnet rotation before addition of nicking endonuclease, which results in rapid uncoiling. (**C**) Schematic of experiments for measuring the time dependence of condensation. PARP1 is added to damaged DNA before flowing through solution containing NAD^+^. (**D**) Force-extension curves showing the change in condensation after adding 400 nM PARP1 followed by adding 1 mM NAD^+^. (**E**) Extension, relative to naked DNA, measured at 0.1-pN force as a function of time after flowing through NAD^+^-containing solution. The graph shows results from two experiments: (i) addition of 1 mM NAD^+^ (*n* = 9 magnetic beads) and (ii) addition of 1 mM NAD^+^ + 10 μM olaparib (*n* = 4). The error bars show SD.

So far, we have focused on the effect of PARP1 binding to undamaged DNA. However, as noted in Introduction, PARP1 is mostly known for its role in DNA damage repair, a process initiated by the synthesis of strongly negatively charged PAR chains at sites of DNA damage. The PAR chains are covalently coupled both to PARP1 itself (automodification) and to other nearby proteins. As the automodified PAR chains grow in size, they are thought to destabilize PARP1-DNA interaction and cause PARP1 to release from DNA (*12*). To investigate this process and its effects on DNA condensation, we extended our investigations to DNA that presented SSBs, introduced by site-specific nicking endonucleases: Nt.BsmAI and Nb.BsmI. Single DNA tethers were selected, and 14 SSBs were made in the 7.9-kbp DNA construct (Fig. 7A). The formation of SSBs was monitored in situ by first checking the topological continuity of the dsDNA tethers by measuring their characteristic shortening in response to supercoiling and plectoneme formation. Then, following endonuclease addition, formation of single-strand nicks (SSBs) allowed the DNA to rapidly relax back to its length at zero turns since the plectonemes unwound via free rotation at the nick sites (Fig. 7B).

After creation of the SSBs, we added 400 nM PARP1 to the solution and measured DNA condensation from the force-extension relationship (Fig. 7, C and D). This resulted in a similar behavior as observed for the undamaged DNA (see Fig. 4A). We next flushed the flow cell with a solution containing NAD^+^ and measured how condensation varied over time by repeating the force-extension curve every minute. Figure 7D shows this change for a buffer solution without PARP1 but containing 1 mM NAD^+^. In Fig. 7E, we quantify the change in condensation by plotting the extension relative to naked DNA at 0.1 pN as a function of time after adding the NAD^+^-containing solution. We found a >70% recovery (i.e., loss of condensation) within 3 min of adding NAD^+^. The release of PARP1 from the DNA and reduction in the condensation effect can be explained by the formation of PAR chains, which are known to reduce PARP1-DNA affinity. We then tested the effect of adding 1 mM NAD^+^ in the presence of 10 μM olaparib and found that DNA remained in its condensed state for the duration of the 10-min experiment and no longer returned to the force-extension behavior characteristic of naked DNA. This behavior is consistent with olaparib competing with NAD^+^, blocking PAR formation and preventing PARP1 from unbinding the DNA. For undamaged DNA, we found a recovery of >70% extension over 8 min when a buffer not containing NAD^+^ was flowed through (fig. S10).

## DISCUSSION

We have used a combination of single-molecule techniques to show that PARP1 condenses both damaged and undamaged DNA. The condensation is reversible by applying a high force (>1 pN). The loss of condensation from application of a step change to high force was observed to occur in a series of discrete changes in extension. The relatively large amplitude of the steps (>100 nm) is not consistent with DNA wrapping around PARP1 but is more easily explained by formation of DNA loops. The wide distribution of step-size amplitudes is also consistent with initial formation by thermal fluctuations and capture by PARP1 binding (*50*). The ~1-pN threshold of condensation is also typical of DNA looping seen in previous studies (*49*). To test the idea that PARP1 can stabilize DNA crossover points, we also performed DNA braiding experiments. These experiments clearly showed that PARP1 forms a bridge between DNA molecules, stabilizing the DNA-DNA intersection as required for a DNA looping activity. Our AFM investigation confirmed that PARP1 binds to undamaged plasmid DNA forming a disordered structure, although individual looping events were not resolved, possibly because of destabilization due to the binding to the AFM substrate. We note that a variety of structures have been observed in electron microscopy and AFM studies of PARP1-DNA interactions such as DNA chaining (*55*), DNA bridging by PARP1 (*56*, *57*), and aggregate formation (*42*) as well as specific binding at DNA damage sites (*42*, *58*).

**Fig. 8:**
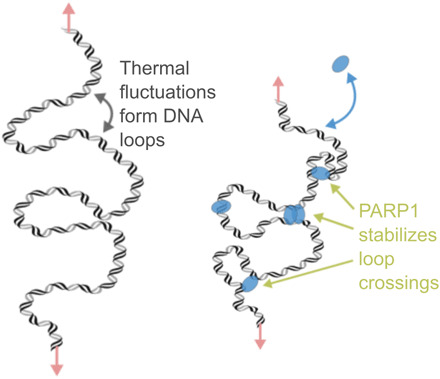
Loop stabilization model for PARP1 condensation of DNA. (**Left**) In the absence of protein and at low forces, thermal fluctuations induce the formation of DNA loops. (**Right**) The multiple DNA binding sites of PARP1 enable bridging to stabilize the loop and thereby reduce the DNA extension.

Figure 8 depicts our model for the DNA condensation activity of PARP1 on a single molecule of intact dsDNA. Thermal fluctuations create intersections of DNA when the tension in the DNA is sufficiently low (below ~1 pN). PARP1 bridges and stabilizes these thermally induced loops, thereby causing a reduction in DNA extension. For single DNA molecules, DNA loop stabilization shows a strong dependence on DNA tension since the probability of loop formation is highly force dependent (*59*, *60*). From the unbinding kinetics measured from the loss of the DNA braid following buffer exchange (Fig. 6D), we infer that the lifetime of the PARP1-DNA bond is on the scale of seconds. This points to there being exchange of PARP1 on undamaged DNA at this time scale even in the condensed state.

The loop stabilization activity of PARP1 is consistent with recent results that show that PARP1 can move through the genome using intersegmental transfer (*25*), since both observations require the use of multiple DNA binding sites. Moreover, our measurement of fast unbinding from DNA braids (Fig. 6D) supports the idea of rapid exchange of PARP1 at intersections between multiple DNA strands. Our experiments, however, do not directly determine the number of PARP1 molecules needed to bridge a single DNA loop. While the crystal structure of PARP1 shows that there are multiple DNA contacts formed through the zinc fingers and WGR domain (*19*), dimers or oligomers of PARP1 could also be formed at the points of loop stabilization. PARP2 has homologous WGR and catalytic domains with PARP1 but lacks the N-terminal zinc finger DNA binding domains. Given that condensation does not occur up to high concentrations (5 μM) of PARP2 nor for a construct of the first two zinc fingers of PARP1, we suggest that condensation is mediated by having both the zinc fingers and WGR, as presented in full-length PARP1.

PARP inhibitors are known to trap PARP1 in an insoluble fraction of chromatin in cellular assays (*10*). The mechanism behind the level of trapping for different inhibitors has been investigated with various in vitro biochemical methods using damaged DNA (*11*, *13*). Since short DNA molecules are used in most binding studies such as fluorescence polarization or surface plasmon resonance, these unavoidably include DNA hairpins or ends, preventing the unambiguous measurement of PARP1 binding to undamaged DNA. Our magnetic tweezers assay allows us to measure real-time PARP1 binding to undamaged DNA since the free ends are outside the measurement region. The results did not show any effect of high concentration of inhibitors on PARP1 condensation of undamaged DNA. When we introduced damage to the DNA through SSBs, we found that the inhibitor olaparib slowed the rate of NAD^+^-induced decondensation. It is known that formation of PAR chains reduces the affinity of PARP1 for DNA breaks by inducing strong electrostatic repulsion (*12*). Our results are consistent with this effect and show that PARylation also affects the ability of PARP1 to condense DNA.

In conclusion, using single-molecule techniques, we have shown that PARP1 condenses DNA by a loop stabilization mechanism. PARP1 is known to play an important role as a chromatin architectural protein together with its role in DNA repair (*61*). Independent of its PARylation activity, it has been observed that PARP1 condenses nucleosome-bound DNA (*62*) and is enriched at regions of heterochromatin (*63*, *64*). In *Drosophila,* PARP1 is also known to inactivate certain transcriptional domains and repress retrotransposable elements (*65*, *66*). Loop stabilization is a novel mechanism that can explain the fundamental physical interaction between DNA and PARP1 that underlies these condensed areas of chromatin. Methods to probe cellular chromatin architecture and protein-induced looping (*67*, *68*) should provide further insight into the importance of DNA looping by PARP1 in the context of the nucleus.

## METHODS

All reagents were from Sigma-Aldrich, UK unless otherwise stated.

### TIRF microscopy

λ-DNA [New England Biolabs (NEB)] was functionalized at one end by annealing with a biotinylated oligonucleotide /5Phos/AGGTCGCCGCCCTTTTT/Bio (IDT) followed by ligation with T4 DNA ligase (NEB). The DNA was purified using a bead purification kit (QIAEX II, QIAGEN). A flow cell was constructed using polydimethylsiloxane (SYLGARD 184, Dow), patterned double-sided tape (AR90880, Adhesive Research), and a glass coverslip (Menzel) coated with 2% nitrocellulose in amyl acetate. The flow cell was incubated for 10 min with streptavidin (1 mg/ml) (NEB) and then 10 min with BlockAid (Thermo Fisher Scientific). Biotinylated λ-DNA was then injected and allowed to bind to the streptavidin-coated surface. Imaging was performed using a buffer of 20 mM Hepes (pH 7.8), 150 mM NaCl, 2 mM MgCl_2_, 0.5 mM tris(2-carboxyethyl)phosphine (TCEP), bovine serum albumin (BSA) (1 mg/ml), β-casein (1 mg/ml), and 500 nM SYTOX Orange (Thermo Fisher Scientific). A constant flow rate of 100 nl/s was applied using a syringe pump (Harvard Instruments PhD 2000). The sample was illuminated by a 532-nm laser in objective TIRF mode with a 60× Nikon objective, and images were acquired at 10 frames per second (fps) with an electron multiplying charge-coupled device camera (Andor iXon).

### Atomic force microscopy

A 496-bp section of λ-DNA was amplified by PCR using Taq polymerase (forward primer, 5′-TGAAATTGCCGCGTATTACGC-3′, reverse primer, 5′-TTTCTCGTAGGTACTCAGTCCG-3′). The PCR product was purified by a kit (QIAquick, QIAGEN). The sequence of the 496-bp product contains a site for Nt.BsmAI between bases 172 and 173, i.e., approximately one-third of the total length. Nicking was performed by incubating the DNA with Nt.BsmAI, and, subsequently, the DNA was further purified (QIAquick, QIAGEN). Relaxed pBR322 plasmid DNA was purchased from Inspiralis Ltd. The block copolymer mPEG_5k_-b-PLKC_10_, methoxy-PEG-block-poly(l-lysine hydrochloride), was purchased as a lyophilized powder from Alamanda Polymers. PLL_150–300k_ [0.1% (w/v), 150,000 to 300,000 molecular weight) was purchased from Sigma-Aldrich.

For AFM imaging, a freshly cleaved mica specimen disk (diameter, 6 mm; Agar Scientific, UK) was functionalized with PLL-PEG, consisting of a mixture of mPEG_5k_-b-PLKC_10_ (1 mg/ml in Milli-Q water) and PLL_150–300k_, prepared as previously described (*40*). The washing and imaging buffer used throughout was 12.5 mM NaCl, 12.5 mM Hepes, and 0.5 mM TCEP (pH 7.8) filtered by passage through 0.2-μm syringe followed by a 10-kDa cutoff centrifugal filter (Amicon Ultra, Millipore). Once the modified mica was prepared, 20 μl of linear 496-bp DNA (1.5 ng/μl) or relaxed pBR plasmid DNA (2.5 ng/μl) was then added to the disk and gently mixed. After a 30-min adsorption, the sample was then washed five times and topped up to 30 μl with imaging buffer. For the preincubation assays, the DNA was incubated with PARP1 (and 500 nM olaparib if stated) for 30 min at room temperature before deposition onto the modified mica surface. Images of PARP1 preincubated with DNA before deposition are shown in Fig. 2 (A and B) and figs. S4C and S5C. For the postincubation assays, after DNA was immobilized and imaged on the modified mica surface, the sample was exposed to the imaging buffer with PARP1 (and 500 nM olaparib if stated). Imaging was resumed after 5-min incubation without washing. See figs. S4A and S5A for images. Despite the differences in the quantity of PARP1 bound to the DNA across these two sample preparation methods, a similar qualitative behavior of PARP1 binding is observed.

All AFM measurements were carried out in buffer and at room temperature. Data were recorded using a Dimension FastScan Bio AFM (Bruker, Santa Barbara, USA), using force-distance curve–based imaging (Bruker’s PeakForce Tapping mode). Force-distance curves were recorded more than 10 to 40 nm (PeakForce Tapping amplitude of 5 to 20 nm) at a frequency of 8 kHz. FastScan D (Bruker) cantilevers were used for all imaging (nominal spring constant of ~0.25 Nm^−1^). Imaging was carried out using PeakForce set points in the range of 15 to 30 mV with a deflection sensitivity of approximately 18 nm/V. Images were recorded at a scan size of 1 to 2 μm with 1024 pixels per line and at a line rate of 4 Hz. Images were processed using either Gwyddion (*69*) or a Python that uses the Gwyddion “pygwy” module, as described in greater detail elsewhere (*70*). All images were initially subjected to correction by first-order line-by-line flattening, median line-by-line flattening, and zeroth-order plane fitting to remove background offset and tilt. The background was also zeroed by setting the mean value to zero. A 1- to 2-pixel (~1 to 2 nm) Gaussian filter was then applied to remove high-frequency noise.

To identify DNA molecules, the Python script was used to find grains that have heights within 1σ of the mean of the height distribution of the image and have a pixel area within 50 to 150% of the median grain area, with the DNA identification verified by direct inspection of the images. The thus identified “grains” were interrogated further to measure the size of PARP molecule(s) bound to linear DNA and the total PARP volume bound per plasmid DNA molecule. To isolate protein molecules bound to DNA, these grains were manually thresholded with a height of ~1.5 times the height of a DNA molecule equivalent to around 2 nm above the background. Volumes were evaluated as the zero-basis volume in Gwyddion, i.e., the volume from zero height covered by the mask. The images used to measure the size of PARP molecule(s) bound to linear DNA were taken using both sample preparation methods described above, where PARP1 is either pre- or postincubated with DNA in the presence or absence of olaparib. The distribution of measured PARP1 volumes bound to linear DNA is visualized as a histogram (Fig. 2D).

### Magnetic tweezers DNA construct

The central part of the DNA construct used for magnetic tweezers was a 7.9-kbp fragment produced by restriction digestion of λ-DNA using Sap I and Bsa I (NEB) and subsequent agarose gel purification. A 478-bp fragment of biotin-labeled DNA was produced by PCR with Taq polymerase (NEB) of λ-DNA using the primers 5′-CGAACTCTTCAAATTCTTCTTCCA-3′ and 5′-GATTGCTCTTCTGTAAGGTTTTG-3′ with a 5:1 ratio of deoxythymidine triphosphate (dTTP):biotin-11-dUTP (deoxyuridine triphosphate) (Jena Bioscience). Similarly, an 878-bp fragment of digoxigenin-labeled DNA was produced by PCR of λ-DNA with primers 5′-TAGTCCAGAACGAGACCGCAACAGCACAACCCAAACTG-3′ and 5′-AATCTGCTGCAATGCCACAG-3′ (underline section indicates overhang) with a 10:1 ratio of dTTP:digoxigenin-11-dUTP (Jena Bioscience). The labeled fragments were purified using a PCR purification kit (QIAquick, QIAGEN). The 478-bp fragment was digested with Sap I, and the 878-bp fragment was digested with Bsa I before each fragment was again purified. The central fragment and two-labeled fragments were then ligated by incubation with T4 DNA ligase (NEB) overnight. The construct was lastly purified by agarose gel electrophoresis. All DNA gel extractions were performed without exposure to ultraviolet or intercalating dyes by staining the outer two lanes only to calculate the band position and using these as alignments for gel excision. The DNA construct length was confirmed by gel electrophoresis (fig. S11). The DNA was conjugated to streptavidin-coated magnetic beads (MyOne T1 Dynabeads; Thermo Fisher Scientific) by incubating for 15 min at room temperature. The concentration of DNA required was empirically determined to maximize the number of single or double tethers.

### Magnetic tweezers measurements

The 1.5-μm-diameter silica microspheres (Bangs Laboratories) were suspended in 2% collodion at approximately 0.05 mg/ml. The solution was then spin-coated onto a glass coverslip to form a thin layer of nitrocellulose with the embedded silica microspheres acting as fiducial markers for drift tracking. A flow cell was formed by cutting channels into double-sided tape (AR90445, Adhesive Research) and sandwiching this between the nitrocellulose-coated coverslip and a second coverslip. A solution (0.1 mg/ml) of anti-digoxigenin antibodies (Sigma-Aldrich) was incubated in the flow cell for 30 min followed by surface passivation for 30 min with BlockAid solution (Thermo Fisher Scientific). After blocking, DNA-labeled magnetic beads were flowed through the chamber and incubated in the flow cell for 10 min. Last, measurement buffer was perfused through the chamber. All measurements were performed in a buffer of 20 mM Hepes (pH 7.8), 150 mM NaCl, 2 mM MgCl_2_, 0.5 mM TCEP, BSA (1 mg/ml), and β-casein (1 mg/ml). Solution exchanges were performed with a custom-built gravity perfusion system at a flow rate of 500 nl/s. To add SSBs, Nt.BsmAI (0.1 U/μl) in 1× CutSmart buffer (NEB) was added to the flow cell and incubated for 5 min followed by Nb.BsmI (0.2 U/μl) in 1× NEB 3.1 buffer (NEB), which was also incubated for 5 min. The flow cell was then flushed with 20 mM Hepes (pH 7.8), 1 M NaCl, and BSA (0.1 mg/ml) before adding the measurement buffer. PARP inhibitors were purchased from Cayman Chemical and diluted in dimethyl sulfoxide (DMSO). Inhibitor experiments used a final concentration of 1% DMSO.

A bright-field microscope combining a high-power light-emitting diode (M660L4-C4, Thorlabs), 60× air objective (Nikon), and camera (DMK 33GX249, Imaging Source) was used to image the magnetic beads. A lookup table for calculating *z* position was created by stepping the objective using a piezo actuator (P721 PiFoc, Physik Instrumente). Bead positions were tracked, in real time, using a previously described algorithm (*71*). The force, *F*, was calibrated according to the formula *F = k*_b_*Tl*/<*x*^2^ >, where *k*_b_ is the Boltzmann constant, *T* is the absolute temperature, *l* is the DNA extension, and <*x*^2^> is a variance in bead *x* position (parallel to the magnetic field lines). A single exponential was used to fit the force versus magnet height relationship. A fast frame exposure time of 0.7 ms was used so that image blurring, due to bead motion, was negligible at the forces used in our experiments (*72*). Images were acquired at 20 fps for all experiments except for PARP1 unbinding traces (Fig. 5), where a frame rate of 200 fps was used. A rank 2 median filter was used for display. Forces were applied to the magnetic beads using a pair of 5-mm cubic neodymium magnets (Supermagnete) mounted on a custom-built *z*-translation stage. A stepper motor coupled to a drive belt enabled rotation of the two magnets at a speed of 1 revolution/s.

### Electromobility gel shift assay

pUC19 plasmid (NEB) was relaxed from its supercoiled form by incubating with Topo I (NEB) and purified (QIAquick, QIAGEN). The relaxed plasmid was then incubated with PARP1 for 10 min in a buffer containing 20 mM Hepes (pH 7.8), 150 mM NaCl, 2 mM MgCl_2_, 0.5 mM TCEP, and BSA (0.05 mg/ml). The DNA complexes were visualized by running on a 1× tris-acetate-EDTA and 1% agarose gel for 1 hour at 70 V and then visualized by staining with GelRed (Biotium).

### PARP1 purification

Recombinant PARP1 carrying an N-terminal hexahistidine AviTag was produced using a pFastBac vector–based baculovirus expression system for expression in Sf21 insect cells. The cells were harvested by centrifugation, and pellets were solubilized in binding buffer [25 mM tris-HCl (pH 7.4), 150 mM NaCl, 1 mM TCEP, and 10 mM imidazole] before sonication in the presence of deoxyribonuclease I (Sigma-Aldrich, D4527) and EDTA-free protease inhibitor cocktail (Roche, 37378900). The lysed pellet was clarified by centrifugation, and the supernatant was mixed with 3 ml of nickel–nitrilotriacetic acid (Ni-NTA) resin (Thermo Fisher Scientific, 88222) and incubated for 1 hour at 4°C. Ni-NTA beads were loaded onto a gravity flow column and washed with binding buffer. PARP1 elution was achieved by using an imidazole gradient in binding buffer. Fractions containing PARP1 were further purified by ion exchange chromatography on a HiTrap Heparin HP (GE Healthcare) column followed by size exclusion chromatography on a HiLoad 16/60 Superdex 200 (Amersham Biosciences) prep grade column. SDS–polyacrylamide gel electrophoresis (PAGE) analysis of the purified PARP1 indicated >90% purity and confirmed PARylation activity in the presence of NAD^+^ and DNA breaks (fig. S11). A *K*_d_ = 71 nM of PARP1 binding to a DNA dumbbell containing a SSB was measured with fluorescence polarization (fig. S11) similar to previous studies (*21*). His-tagged Zn1Zn2 (first two zinc fingers of PARP1) was expressed using a cell-free expression system (NEBExpress). RNA was first transcribed and purified from the plasmid (NEB T7 Quick High Yield RNA Synthesis Kit and NEB Monarch RNA cleanup). The RNA was then used directly in the NEBExpress reaction. The reaction was incubated for 3 hours and then quenched with ribonuclease A (0.5 mg/ml) (NEB) before purification using Ni-NTA resin (NEBExpress Ni-NTA spin columns) and desalting using Zeba resin (Thermo Fisher Scientific). The reaction products were confirmed by SDS-PAGE gel (fig. S10).

## SUPPLEMENTARY MATERIALS

Supplementary material for this article is available at http://advances.sciencemag.org/cgi/content/full/7/33/eabf3641/DC1

## Appendix

View/request a protocol for this paper from *Bio-protocol*.
